# M2 macrophage-derived exosomes and miR-132-3p promote gastric cancer cell aggressiveness and attenuate ferroptosis-related changes

**DOI:** 10.3389/fonc.2026.1934498

**Published:** 2026-09-28

**Authors:** Tao Zhang, Yong Huang, Peng Cheng, Songhan Qin, Yifan Cao, Ming Xie, Jiwei Wang

**Affiliations:** 1 Department of General Surgery, Digestive Disease Hospital, Affiliated Hospital of Zunyi Medical University, Zunyi, China; 2 Guizhou Provincial Key Laboratory of Digestive Disease, Zunyi, China

**Keywords:** exosome, ferroptosis, gastric cancer, MiR-132-3p, tumor microenvironment

## Abstract

**Purpose:**

This study aimed to explore the correlation between the ability of M2-type tumor-correlated macrophage-derived exosomes (EXOs) to trigger the aggressiveness of gastric cancer (GC) cells and the miR-132-3p expression.

**Methods:**

EXOs were isolated from the conditioned media of M0 and M2 macrophages and characterized by transmission electron microscopy and Western blotting. miR-132-3p expression was determined by qRT-PCR. HGC-27 cells were divided into the control, miR-132-3p mimic, miR-132-3p inhibitor, M0-EXO, and M2-EXO groups. Cell viability, proliferation, migration, and invasion were assessed using CCK-8, EdU, and Transwell assays. Intracellular GSH, SOD, MDA, and iron levels were measured using colorimetric assays. FTH1, LIFR, and GPX4 expressions were detected by Western blotting. RNA sequencing and bioinformatics analyses were performed to investigate miR-132-3p-mediated transcriptional changes and ferroptosis-related pathways in HGC-27 cells.

**Results:**

miR-132-3p was significantly enriched in M2 macrophage-derived exosomes and was highly expressed in GC, with its expression associated with tumor stage, grade, and other clinicopathological features. M2-Exo treatment significantly increased miR-132-3p levels in HGC-27 cells. Both miR-132-3p overexpression and M2-Exo treatment enhanced the proliferation, migration, and invasion of HGC-27 cells, and were accompanied by reduced intracellular iron and MDA levels, increased GSH content and SOD activity, upregulated FTH1 and GPX4 expression, and decreased LIFR expression. In contrast, miR-132-3p inhibition produced largely opposite effects. RNA-seq identified 275 differentially expressed genes following miR-132-3p overexpression, including 216 upregulated and 59 downregulated genes. GO and KEGG analyses revealed significant alterations in biological processes and pathways related to oxygen transport, ATP synthesis, oxidative phosphorylation, thermogenesis, spliceosome function, and endoplasmic reticulum protein processing. Integration of the RNA-seq-derived DEGs with FerrDb identified 12 ferroptosis-related DEGs, including 11 upregulated genes and 1 downregulated gene. qRT-PCR validation showed expression patterns broadly consistent with the RNA-seq results.

**Conclusion:**

miR-132-3p expression in the EXOs of M2-type macrophages was significantly elevated, and the EXOs of M2-type macrophages significantly triggered the aggressiveness of GC cells and inhibited ferroptosis in GC cells, which may be resulting from the prevention of ferroptosis through the transport of miR-132-3p; nevertheless, the specific mechanism of action needs to be further elucidated.

## 1 Introduction

Gastric cancer (GC) has a silent onset, and thus, its diagnosis is often at an advanced stage, with most patients already experiencing distant metastases. GC severely affects the survival quality and treatment outcomes of patients (1). As per the National Cancer Center, 359,000 new cases of GC were found nationwide in 2022; thus, it is the fifth most prevalent malignancy. Additionally, 260,000 new GC-related deaths were found; thus, it is the third largest cause of cancer-related deaths (2). Over 50% of patients may experience recurrence even after radical resection, and those with recurrent GC have a very high death risk. Early detection, diagnosis, and treatment are the best approaches for reducing mortality related to GC (3). Tumor-associated macrophages (TAMs), major elements of the tumor microenvironment (TME), exhibit an M2 macrophage phenotype. These cells can trigger the aggressiveness and drug resistance of GC cells by secreting cytokines, exosomes (EXOs), and other factors, thus triggering the progression of GC (4–6). Changes in exosomal miRNAs may act as serum diagnostic biomarkers for GC (7).

miR-132-3p expression is significantly elevated in GC and has been closely associated with enhanced tumor cell proliferation and malignant progression (8). As an important epigenetic regulator, miR-132-3p can modulate the expression of multiple downstream target genes involved in cell survival, metabolism, and stress responses, suggesting that its aberrant expression may contribute to GC development through multiple mechanisms. In addition to its intracellular functions, miR-132-3p can also be transferred between cells through extracellular vesicles. Notably, M2-polarized macrophages have been reported to secrete exosomes (EXOs) enriched in miR-132-3p, thereby regulating recipient-cell functions and promoting tissue repair in myocardial infarction (9). Given that tumor-associated macrophages, particularly M2-like macrophages, represent a major component of the GC tumor microenvironment and actively communicate with cancer cells through exosomal miRNAs, it is reasonable to speculate that macrophage-derived exosomal miR-132-3p may also participate in the regulation of GC cell fate.

Ferroptosis is an iron-dependent form of regulated cell death characterized by excessive intracellular iron accumulation, lipid peroxidation, depletion of antioxidant defenses, and distinctive mitochondrial morphological alterations (10). Accumulating evidence indicates that ferroptosis is closely involved in the initiation, progression, and therapeutic response of GC (11–13). Induction of ferroptosis can suppress GC cell viability and tumor growth, whereas enhanced resistance to ferroptotic stress may provide cancer cells with a survival advantage under adverse conditions (14). Importantly, ferroptosis is tightly controlled by multiple metabolic and signaling networks that can also be regulated by miRNAs.

Therefore, whether M2 macrophage-derived exosomal miR-132-3p influences ferroptosis-related pathways in GC cells and thereby contributes to tumor progression remains an important question that warrants further investigation. Therefore, herein, we focused on M2 macrophage-derived EXOs and miR-132-3p to assess their effects on the aggressiveness of GC cells, as well as their correlation with ferroptosis, to preliminarily assess the potential of exosomal miR-132-3p as a diagnostic target for GC.

## 2 Materials and methods

### 2.1 Reagents

RPMI-1640 (11875119) was acquired from Gibco (Waltham, USA). Phorbol 12-myristate-13-acetate (PMA) (P1585) was acquired from Sigma-Aldrich (St. Louis, USA). IL-4 (11846-HNAE) and IL-13 (10369-HNAC) were acquired from Sino Biological (Beijing, China). Rabbit anti-leukemia inhibitory factor receptor (LIFR) (22779–1-AP), mouse anti-CD81 (66866-1-Ig), rabbit anti-TSG101 (28283-1-AP), rabbit anti-Tubulin (80762-1-RR), rabbit anti-Calnexin (10427-2-AP), rabbit anti-CD9 (20597-1-AP) were acquired from Proteintech (Wuhan, China). Rabbit anti-ferritin heavy chain 1 (FTH1) (DF6278) and rabbit anti-glutathione peroxidase 4 (GPX4) (DF6701) were purchased from Affinity (Liyang, China). EXO extraction kits (76064) were purchased from QIAGEN (Hilden, Germany). EdU detection kits (C0071S) and RIPA lysis buffer (P0013B) were acquired from Beyotime Biotechnology Co., Ltd (Shanghai, China). Reverse transcription kits (AE311) and SYBR Green PCR kits (AQ131) were acquired from TransGen Biotech (Beijing, China). Malondialdehyde (MDA) content detection kits (BC0025), reduced glutathione (GSH) content detection kits (BC1170), and superoxide dismutase (SOD) activity detection kits (BC5165) were acquired from Solarbio (Beijing, China). Lipofectamine 2000 (11668-019) was purchased from Invitrogen (Carlsbad, USA). Mouse anti-GAPDH (GB12002-100) was bought from Servicebio (Wuhan, China). BCA protein concentration assay kits (PC0020) were purchased from Solarbio (Beijing, China). The hsa-miR-132-3p mimic (miR10000426-1-5), hsa-miR-132-3p inhibitor (miR20000426-1-5), micrON mimic NC (miR1N0000001-1-5) and micrOFF inhibitor NC (miR2N0000001-1-5) were bought from RiboBio (Guangzhou, China).

### 2.2 Cell culture

Human GC cell lines, HGC-27 (SCSP-5263), MKN-45 (SCSP-5473), NCI-N87 (SCSP-534) and MKN74 (SCSP-5540), human gastric epithelial cell line (GES-1) (GNHu65) and human monocytic leukemia cell line (THP-1) (SCSP-567) were acquired from the Cell Bank of the Typical Culture Collection of the Chinese Academy of Sciences (Shanghai, China). MKN-45, NCI-N87, HGC-27, MKN74, THP-1, and GES-1 cells were cultivated in RPMI 1640 medium with 10% fetal bovine serum and 1% (100 U/mL penicillin, 100 µg/mL streptomycin) at 37 °C in 5% CO_2_.

THP-1 cell culture and induction THP-1 cells were cultivated in RPMI-1640 medium enriched with 10% fetal bovine serum (FBS) and 1% penicillin-streptomycin. The cells were stimulated with 150 nmol/L PMA for 24h to trigger their differentiation into M0 macrophages. After eliminating the medium, the cells were PBS-rinsed and exposed to IL-4/13 (both 20 ng/mL) for 48 h to trigger M2 macrophages.

### 2.3 Bioinformatics analysis

Differentially expressed genes (DEGs) were estimated via the R “edgeR” with the Gene Expression Omnibus (GEO) database, specifically GSE97467 (M2 macrophage EXO miRNA expression data). The selection criteria were |logFC| > 2 and *P* < 0.05. The miR-132-3p expression and its correlation with the clinical pathology of GC were estimated via the UALCAN (http://ualcan.path.uab.edu/index.html).

### 2.4 EXO extraction from macrophage culture medium

The supernatant (4 mL) from M0 and M2 macrophage cultures was collected. EXOs were extracted from M0 and M2 macrophage culture media using an EXO extraction kit per the manufacturer’s protocols. We resuspended the EXO pellet in 10 μmol/L PKH67 solution, and a 30-min incubation was conducted at 37 °C. The labeled EXO pellet was rinsed by ultracentrifugation to eliminate unbound dye. Then, we resuspended the pellet in 1 mL of PBS and gently mixed it.

### 2.5 EXO scanning electron microscopy

We fixed EXOs with 2% glutaraldehyde (prepared in 0.1 mol/L PBS [pH 7.4]). We placed the fixed EXOs on poly-L-lysine-treated 100-mesh carbon-coated and poly(methyl methacrylate)-coated nickel grids. Post-incubation for 30 min, the grids were PBS-rinsed, and incubation was conducted in 1% glutaraldehyde for 5 min. After rinsing the samples with deionized water, they were stained with 4% uranyl acetate, filtered through a membrane, and incubated for 5 min. We eliminated excess liquid with filter paper, and the samples were dried. We visualized the samples via a scanning electron microscope (80 kV).

### 2.6 HGC-27 cell transfection and grouping

HGC-27 cells (1×10^6^/well) were cultivated in six-well plates and classified into these groups in a random manner: Control, OV-miR-132-3p (miR-132-3p mimics treatment group), In-miR-132-3p (miR-132-3p inhibitor treatment group), M0-Exo (M0 macrophage-derived exosome treatment group), and M2-Exo (M2 macrophage-derived exosome treatment group). The control group was cultivated normally without intervention. We transfected the OV-miR-132-3p and In-miR-132-3p groups with the miR-132-3p mimic and the miR-132-3p inhibitor, respectively, at final concentrations of 50 and 100 nmol/L. The M0-Exo and M2-Exo groups were subjected to EXOs from M0 and M2 macrophages, respectively. Before treatment of HGC-27 cells, the protein concentrations of the isolated exosomes were determined using a BCA protein assay kit, and the M0-Exo and M2-Exo preparations were adjusted to the same protein concentration to ensure equivalent exosomal protein doses between the two groups. OV-NC and In-NC represent HGC-27 cells treated with micrON mimic NC and micrOFF inhibitor NC, respectively. The cells in all groups were cultivated for 48 h before further analysis was conducted.

### 2.7 EXO uptake experiments

PKH67-labeled M2 macrophage-derived EXOs were applied to HGC-27 cells and incubation was conducted for 48 h. The uptake of EXOs by HGC-27 cells was visualized via fluorescence microscopy.

### 2.8 qRT-PCR detection

Total RNA isolation from M0-Exos, M2-Exos, and HGC-27 cells was conducted via TRIzol reagent. After cDNA synthesis was conducted via a cDNA synthesis kit, we conducted qRT-PCR. This is the PCR program: 94 °C for 30 s, and then 40 cycles of 94 °C for 5 s, 61 °C for 35 s, and a melt curve (95 °C for 10 s, 65 °C for 60 s, and 97 °C for 1 s). The differences in the target genes’ expression were analyzed via the 2^-ΔΔCT^ technique. qRT-PCR primers’ design and synthesis were conducted by Wuhan Lingjiesi Biotechnology Co., Ltd (Wuhan, China). **Table 1** displays the primer sequences.

**Table 1:** Primer sequences.

Gene	F (5’–3’)	R (5’–3’)
miR-132-3p-RT	GTCGTATCCAGTGCAGGGTCCGAGGTATTCGCACTGGATACGACCGACCA	
miR-132-3p	AGCCAGCGTAACAGTCTACAGC	TAACAGTCTACAGCCATGGTCG
U6	CTCGCTTCGGCAGCACA	AACGCTTCACGAATTTGCGT
WIPI1	GTGGAGCCGGCGCGGGCGGGCTGCT	ACTCCATTCTTCCGCCTTCC
HSPA5	CATCACGCCGTCCTATGTCG	CGTCAAAGACCGTGTTCTCG
HERPUD1	ATGGAGTCCGAGACCGAAC	TTGGTGATCCAACAACAGCTT
ETV4	GATGAAAGCCGGATACTTGGAC	TTCGCGCAAGCTCCCATTT
NDRG1	AAACCTCGCCTGGCTCCCAGCTGGT	CGAGGGCGGATGGTGAACTG
IDH1	TGTGGTAGAGATGCAAGGAGA	TTGGTGACTTGGTCGTTGGTG
HNF4A	CACGGGCAAACACTACGGT	TTGACCTTCGAGTGCTGATCC
HBA1	ACTCTTCTGGTCCCCACAGACTCAG	CTGAGTCTGTGGGGACCAGAAGAGT
ENPP2	ACTTTTGCCGTTGGAGTCAAT	GGAGTCTGATAGCACTGTAGGA
HILPDA	AAGCATGTGTTGAACCTCTACC	TGTGTTGGCTAGTTGGCTTCT
SAT1	AGACCATTTCCTACAACTATGGCTT	ATGACTAATACAAACGGCTC
BLOC1S5-TXNDC5	GATGAGTGGCGGAGGGACAGAGACC	GCCCACCCGCGGCCACGCTGCGCCT

### 2.9 CCK-8 assay for cell viability

We cultivated HGC-27 cells in five groups with interventions. Post-treatment, CCK-8 solution was applied (10 μL/well), and incubation was conducted at 37 °C for 4 h. A microplate reader was utilized to read the absorbance (OD value) at 450 nm.

### 2.10 5-Ethynyl-2’-deoxyuridine assay

HGC-27 cells (1×10^6^/well) were cultivated in six-well plates and treated as per the five intervention groups. The EdU assay was conducted as per the manufacturer’s protocols, and the quantity of EdU-positive cells was assessed to estimate cell growth.

### 2.12 Transwell assay for cell invasion and migration assay

HGC-27 cells (1×10^5^/well) were cultivated in 24-well plates and treated as per the five intervention groups. We added 600 μL of complete medium with 10% FBS to each well, and a Transwell chamber (8 μm pore size) PET membrane (Falcon) was positioned in each well. This process was performed gently to avoid the formation of bubbles between the bottom of the chamber and the culture medium. Next, 200 μL of the previously prepared transfection reagent-cell mixture was applied to the top chamber of each well, and incubation was conducted. After 48 h, the cell culture plate was detached, and the chambers were taken out. The top medium was eliminated, and the top layer of cells was smoothly eliminated with a wet cotton swab. After a 15-min fixation of cells with 4% paraformaldehyde, staining was conducted with 0.1% crystal violet solution for 20 min. We counted cells in each field of view under an inverted microscope (100×), and images were captured and saved.

Matrigel was thawed on ice, and we diluted the original gel 1:8 with a serum-free medium. Subsequently, 80 μL of the Matrigel solution was spread equally on the top layer of the PET membrane. After incubation for 1 h, the Matrigel solution was aspirated, and the prepared transfection reagent-cell mixture was applied. The remaining steps were identical to those for the migration trial.

### 2.13 Detection of GSH, MDA and SOD levels

HGC-27 cells were classified into five groups and treated accordingly. After the intervention, we collected the cells from each group, sonicated them, and spun them at 10,000 r/min for 15 min at 4 °C. The supernatant collection was conducted, and the protein level was measured via a BCA protein assay kit. Alterations in the MDA, GSH, and SOD levels were determined following the procedures outlined in the GSH, MDA, and SOD assay kits.

### 2.14 Iron ion content detection

HGC-27 cells were classified into five groups and treated accordingly. After the intervention, we collected the cells, sonicated them, and spun them at 3,000 r/min for 20 min. The supernatant collection from each group was conducted. The changes in iron ion levels in the cells from each group were assessed via a cell iron content detection kit.

### 2.15 Western blotting

Total proteins were extracted by using RIPA buffer. The total protein concentration was assessed by using BCA kit, and we loaded 30 μg of protein into each well. We conducted SDS-PAGE at 80 V for 30 min, and then 120 V for 2 h, after which we transferred the proteins to a PVDF membrane. An incubation of membrane was conducted overnight at 4 °C with these primary antibodies: mouse anti-CD81 (1:4000), rabbit anti-TSG101 (1:4000), rabbit anti-Tubulin (1:30000), rabbit anti-Calnexin (1:10000), rabbit anti-CD9 (1:4000), rabbit anti-LIFR (1:1000), rabbit anti-FTH1 (1:500), rabbit anti-GPX4 (1:800) and mouse anti-GAPDH (1:20000). After rinsing with TBST three times (5 min each), an HRP-conjugated anti-rabbit/mouse secondary antibody was applied. After the ECL substrate was added, the membranes were scanned using a fully automated chemiluminescence analyzer. The protein band intensity for each group was analyzed using the ImageJ2X software.

### 2.16 Transcriptome sequencing analysis

RNA-seq was performed using the DNBSEQ-T7 platform by BGI, followed by bioinformatics analysis. We conducted differential expression analysis via the DESeq2 software (selection criteria: |FoldChange| ≥ 2, *P* ≤ 0.05). We conducted Kyoto Encyclopedia of Genes and Genomes (KEGG), Gene ontology (GO), and gene set enrichment analysis (GSEA) via the clusterProfiler software, with thresholds of p-adjust (FDR) < 0.05. DEGs were analyzed along with the ferroptosis-related database FerrDb (http://www.zhounan.org/ferrdb/current/operations/download.html) to examine the effect of miR-132-3p on the ferroptosis-related genes’ expression in GC cells.

### 2.17 Nanoparticle tracking analysis

The particle size distribution and concentration of macrophage-derived exosomes were determined by nanoparticle tracking analysis (NTA) using a ZetaView nanoparticle tracking analyzer (PMX-120; Particle Metrix, Germany). Prior to measurement, the instrument was calibrated using 100-nm polystyrene beads (Cat. No. 3100A; Thermo Fisher Scientific, USA). Exosome samples were diluted in 1× PBS (Servicebio, Wuhan, China; Cat. No. G4202-500ML) to an appropriate concentration and analyzed using a 520-nm laser in scatter mode. Particle size distribution and concentration were analyzed using ZetaView software (version 8.05.14 SP7) and expressed as particle diameter (nm) and particles/mL, respectively.

### 2.18 Statistical analysis

GraphPad Prism 6.0 was employed for data analysis. All trials were conducted in 3 repeats, and data were reported as the mean ± standard deviation (SD). We conducted one-way ANOVA to estimate multiple-group variations, whereas the LSD*-t* test was conducted to determine two-group variations. *P* < 0.05 was deemed significant.

## 3 Results

### 3.1 miR-132-3p is highly expressed in M2 macrophage-derived Exos and GC

The GEO (GSE97467) analysis revealed that miR-132-3p was significantly overexpressed in EXOs derived from M2 macrophages (log2FoldChange = 6.15, *P* < 0.01) (**Figure 1A**). TCGA analysis illustrated that miR-132-3p was overexpressed significantly in patients with GC and was strongly correlated with clinicopathological factors such as stage, metastasis and grade (**Figures 1B–E**). The results of the qRT-PCR analysis, in which the human gastric mucosal epithelial cell line (GES-1) was employed as a control, illustrated a significant elevation in the miR-132-3p in GC cells (*P* < 0.01), with the highest expression observed in HGC-27 cells, which were chosen for further experiments (**Figure 1F**).

**Figure 1:**
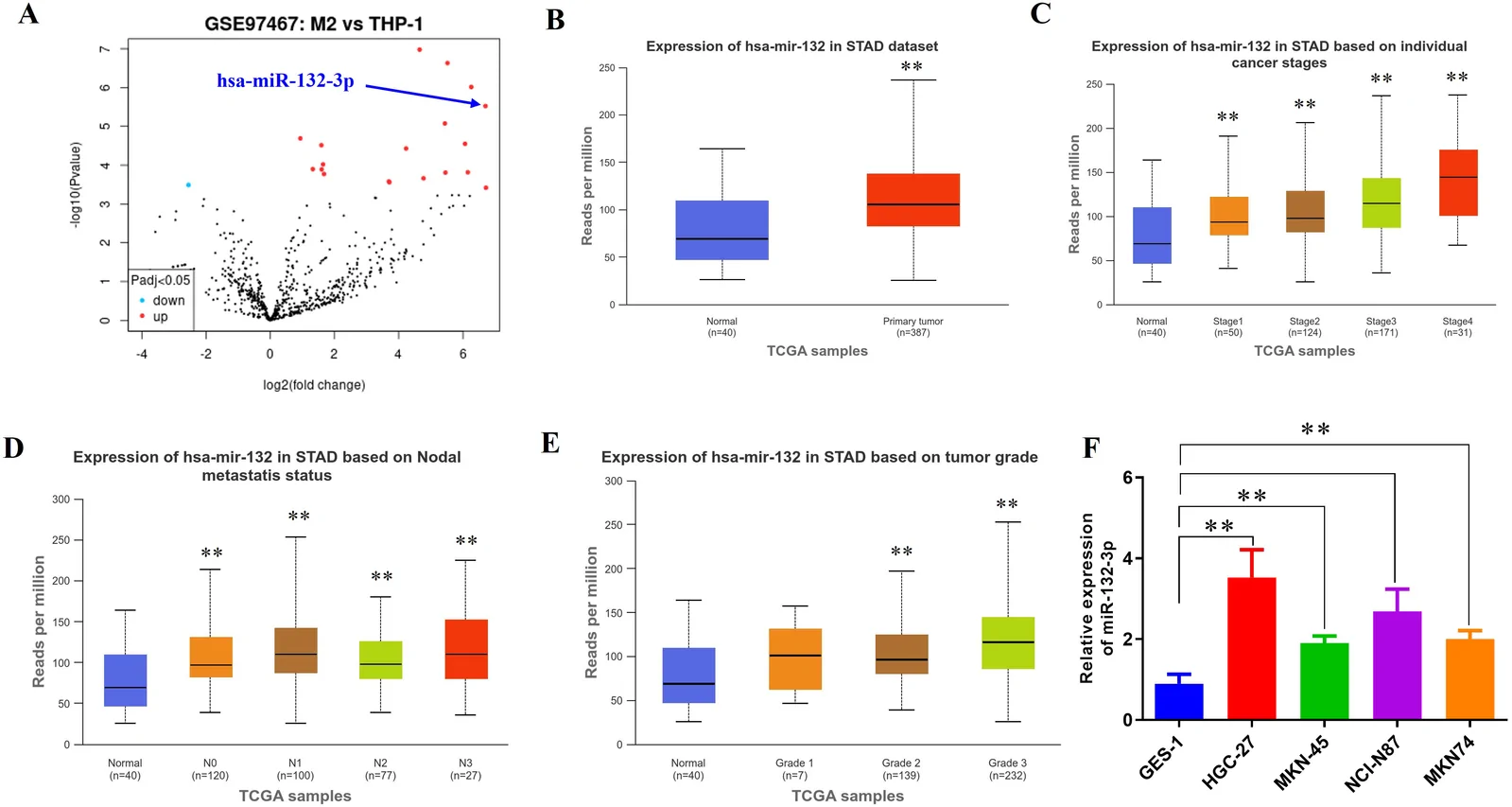
miR-132-3p expression in M2 macrophages and its relationship to the clinicopathology of GC. **(A)** Differential miRNA expression in exosomes derived from M2 macrophages and THP-1 cells was analyzed using the GEO database. **(B–E)** TCGA of the miR-132-3p expression in GC **(B)** and its relationship with grade **(C)**, metastasis **(D)** and stage **(E)**. **(F)** Differential expression of miR-132-3p among different GC cell lines. ^**^P < 0.01 vs. Normal.

### 3.2 M2 macrophage-derived Exos and miR-132-3p promote HGC-27 cell proliferation

Electron microscopy analysis revealed that EXOs were extracted from M2 macrophages (M2-Exos) (**Figure 2A**). Moreover, significant expression of the exosomal markers (CD81 and CD63) validated the successful isolation of EXOs (**Figure 2B**). Additionally, the exosomal marker CD9 was enriched in M2-Exo, whereas the endoplasmic reticulum marker Calnexin was detected in M2 macrophages but was absent or markedly reduced in M2-Exo, supporting the purity of the isolated exosome preparations (**Figure 2C**). NTA further showed that the isolated exosomes displayed a nanoscale particle size distribution, with a median diameter (X50) of 123.2 nm, a mean diameter of 133.1 nm, and a peak diameter of 128.1 nm(**Figure 2D**). These findings further supported the successful isolation and characterization of M2 macrophage-derived exosomes. Uptake experiments revealed that HGC-27 cells could absorb M2-Exos (**Figure 2E**).

**Figure 2:**
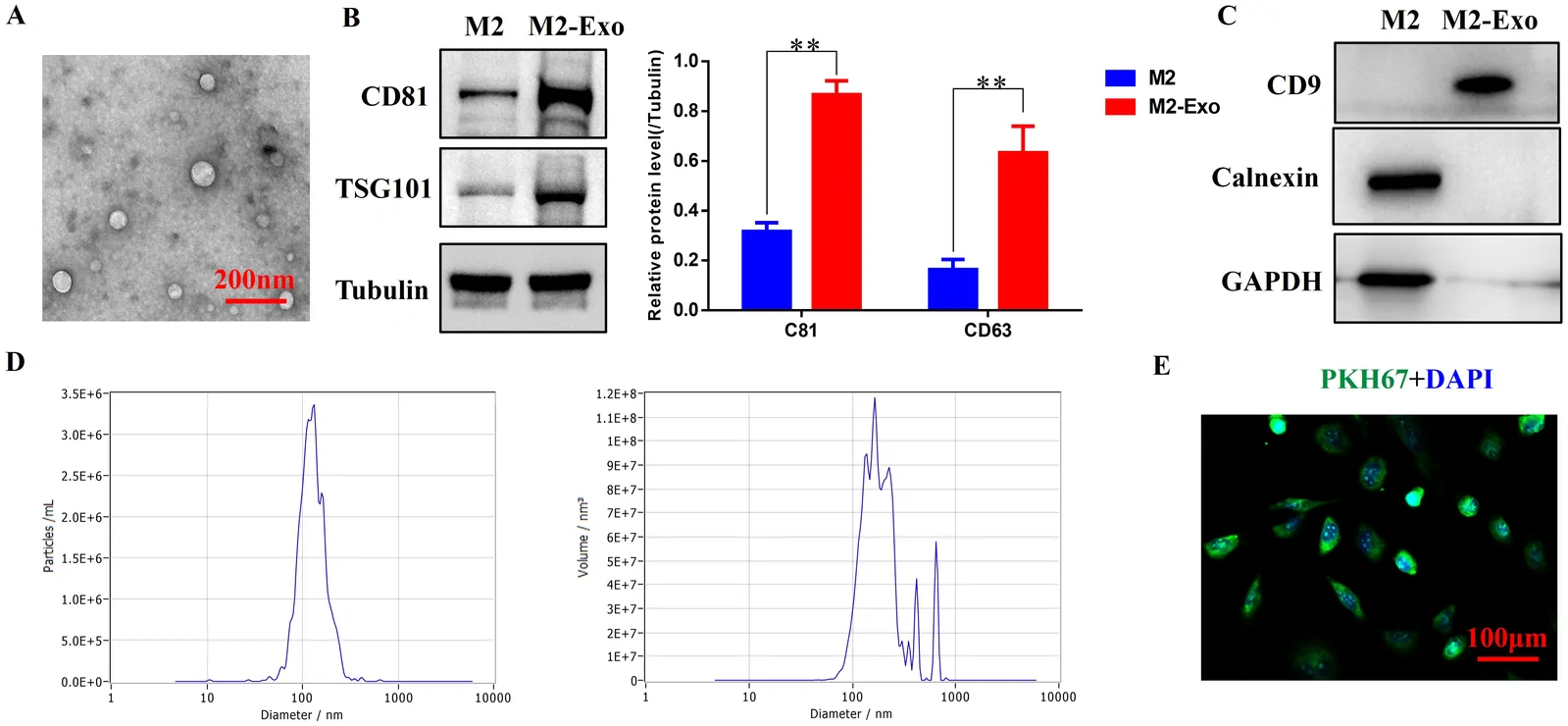
Identification of M2-Exos. Identification of M2-Exos. **(A)** Representative scanning electron microscopy images showing EXO morphology. **(B, C)** Western blotting of exosomal markers. **(D)** NTA particle size distribution. Median diameter (X50): 123.2 nm, Mean diameter: 133.1 nm, Particle concentration: 2.0 × 10^11^ particles/mL. **(E)** Uptake of EXOs by HGC-27 cells. **P < 0.01.

Further, miR-132-3p expression increased significantly in M2-Exos (*P* < 0.01) (**Figure 3A**). The miR-132-3p mimics and M2-Exos significantly promoted miR-132-3p expression in HGC-27 cells, while the miR-132-3p inhibitor significantly suppressed its expression (*P* < 0.01) (**Figure 3B**). The miR-132-3p mimics and M2-Exos significantly elevated the viability and growth of HGC-27 cells, but the miR-132-3p inhibitor significantly declined their viability and inhibited their proliferation (*P* < 0.01) (**Figures 3C–E**).

**Figure 3:**
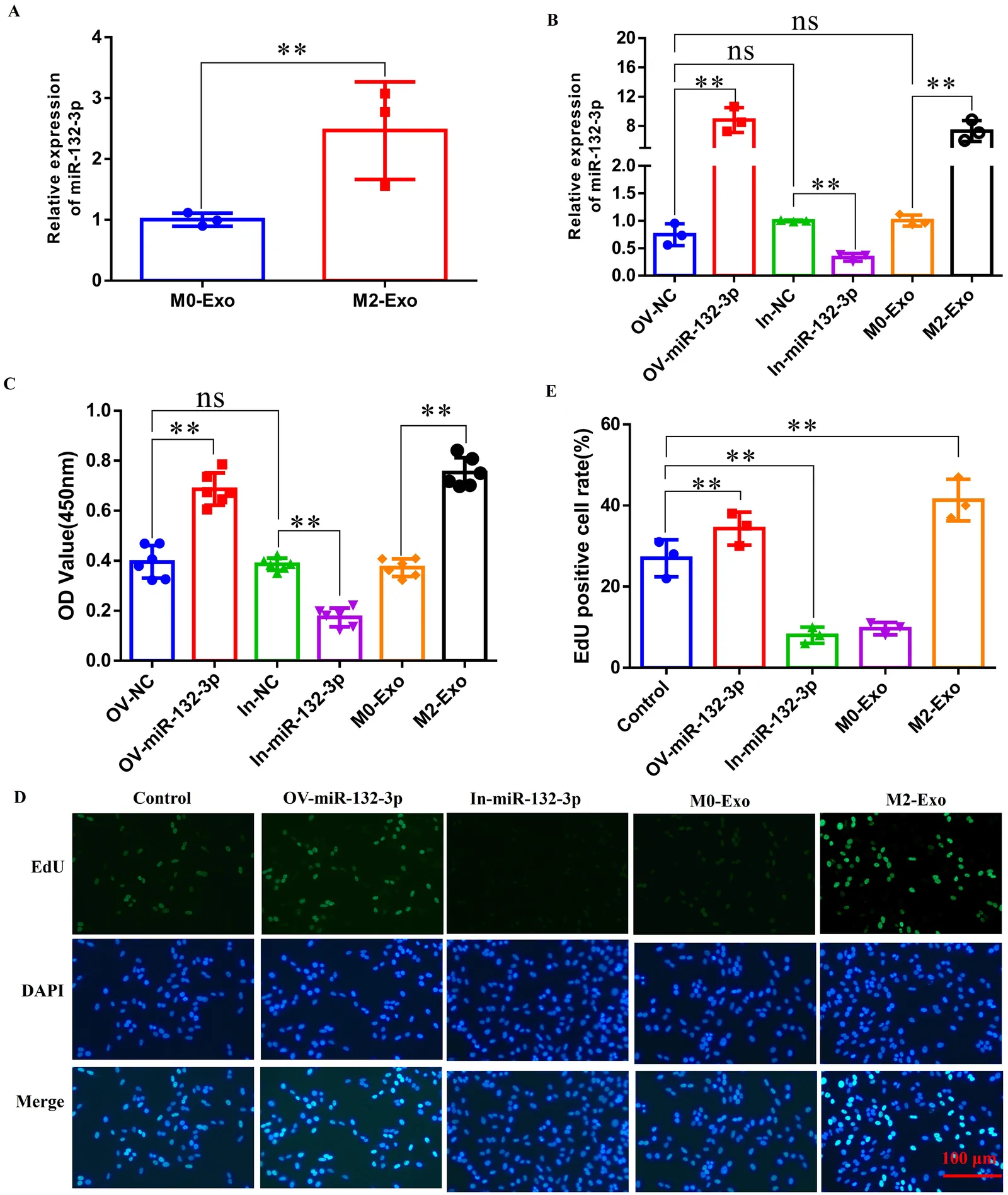
miR-132-3p expression in M2 macrophage-derived EXOs and its effects on HGC-27 cell proliferation. **(A, B)** qRT-PCR of miR-132-3p expression in EXOs **(A)** and HGC-27 cells **(B)**. **(C–E)** miR-132-3p effects on HGC-27 cell growth assessed by CCK-8 **(C)** and EdU assays **(D, E)**. ^ns^*P* > 0.05, ^**^P < 0.01.

### 3.3 M2 macrophage-derived Exos and miR-132-3p promote HGC-27 cell migration and invasion

Transwell assays showed that miR-132-3p mimic and M2-Exos significantly promoted the migration and invasion of HGC-27 cells (*P* < 0.01). In contrast, the miR-132-3p inhibitor markedly suppressed HGC-27 cell migration and invasion (*P* < 0.01) (**Figure 4**).

**Figure 4:**
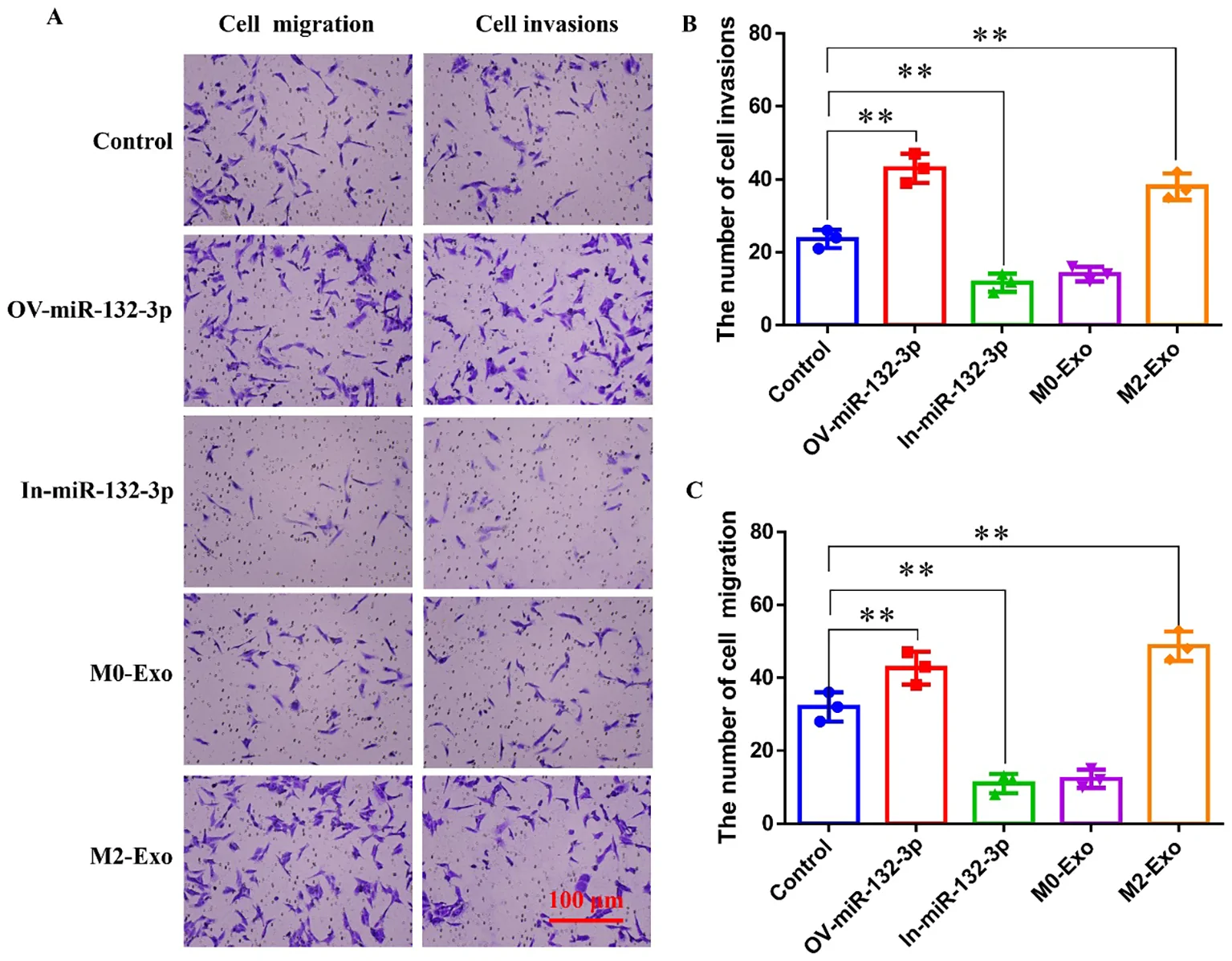
Effects of miR-132-3p and macrophage-derived exosomes on the migration and invasion of HGC-27 cells. **(A)** Representative images of HGC-27 cell invasion and migration. **(B)** Quantification of HGC-27 cell invasion in different treatment groups. **(C)** Quantification of HGC-27 cell migration in different treatment groups. ^**^P < 0.01.

### 3.4 M2 macrophage-derived Exos and miR-132-3p reduce intracellular iron levels in HGC-27 cells

The miR-132-3p mimics and M2-Exos significantly reduced intracellular iron levels in HGC-27 cells, but the miR-132-3p inhibitor significantly increased intracellular iron levels in HGC-27 cells (*P* < 0.01) (**Table 2**).

**Table 2:** The iron ion content in HGC-27 cells.

Group	Iron ion content in protein (ng/mg)
Control	5892.79 ± 186.88
OV-miR-132-3p	4680.17 ± 63.25**
In-miR-132-3p	8134.12 ± 179.91**
M0-Exo	7502.19 ± 190.39
M2-Exo	2532.99 ± 238.27**

Control: HGC-27 cells cultured under normal conditions without treatment with miR-132-3p mimic, miR-132-3p inhibitor or exosomes. OV-miR-132-3p: HGC-27 cells treated with miR-132-3p mimic. In-miR-132-3p: HGC-27 cells treated with miR-132-3p inhibitor. M0-Exo, HGC-27 cells treated with exosomes derived from M0 macrophages. M2-Exo, HGC-27 cells treated with exosomes derived from M2 macrophages.

^**^ *P* < 0.01 vs. Control.

### 3.5 M2 macrophage-derived Exos and miR-132-3p alter ferroptosis-related biochemical parameters

The miR-132-3p mimics and M2-Exos significantly diminished the MDA content (*P* < 0.01), elevated the GSH content and SOD activity (*P* < 0.01) in HGC-27 cells. Conversely, the miR-132-3p inhibitor significantly elevated the MDA content (*P* < 0.01) and inhibited the GSH content and SOD activity (*P* < 0.01) in HGC-27 cells (**Table 3**).

**Table 3:** GSH and MDA content, and SOD activity in HGC-27 cells.

Group	GSH (μmol/gprot)	MDA (nmol/mgprot)	SOD (U/mgprot)
Control	665.19 ± 2.62	1.36 ± 0.36	131.38 ± 0.51
OV-miR-132-3p	873.40 ± 1.62**	0.62 ± 0.10**	283.39 ± 21.53**
In-miR-132-3p	481.51 ± 1.67**	3.19 ± 0.07**	44.62 ± 1.39**
M0-Exo	644.49 ± 1.19	1.56 ± 0.28	128.81 ± 9.79
M2-Exo	1028.09 ± 58.14**	0.43 ± 0.08**	386.16 ± 27.34**

Control: HGC-27 cells cultured under normal conditions without treatment with miR-132-3p mimic, miR-132-3p inhibitor or exosomes. OV-miR-132-3p: HGC-27 cells treated with miR-132-3p mimic. In-miR-132-3p: HGC-27 cells treated with miR-132-3p inhibitor. M0-Exo, HGC-27 cells treated with exosomes derived from M0 macrophages. M2-Exo, HGC-27 cells treated with exosomes derived from M2 macrophages.

^**^ *P* < 0.01 vs. Control.

Collectively, these biochemical alterations were consistent with reduced ferroptotic stress in HGC-27 cells following miR-132-3p overexpression or M2-Exo treatment.

### 3.6 M2 macrophage-derived Exos and miR-132-3p regulate ferroptosis-related proteins

The miR-132-3p mimics and M2-Exos significantly upregulated the protein expression levels of FTH1 and GPX4 (*P* < 0.01) and inhibited the protein expression level of LIFR (*P* < 0.01) in HGC-27 cells. Conversely, the miR-132-3p inhibitor significantly inhibited the protein expression levels of FTH1 and GPX4 (*P* < 0.01), and upregulated the protein expression level of LIFR (*P* < 0.01) in HGC-27 cells (**Figure 5**).

**Figure 5:**
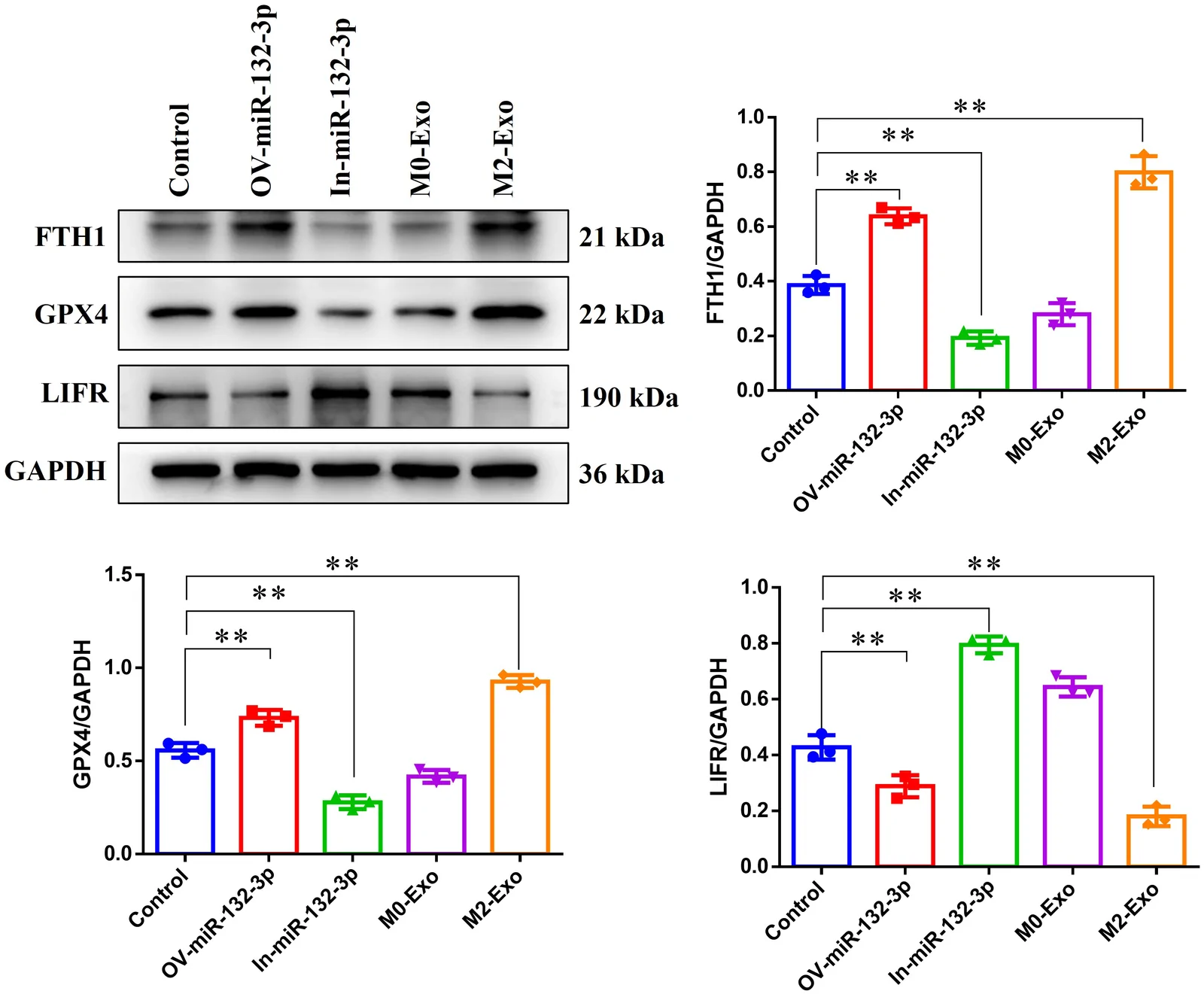
Western blotting was used to detect the expression of FTH1, GPX4 and LIFR in HGC-27 cells. ^**^P < 0.01.

### 3.7 Transcriptomic profiling of HGC-27 cells following miR-132-3p overexpression

The RNA-seq analysis illustrated that miR-132-3p mimics significantly upregulated 216 genes expression and downregulated 59 genes expression in HGC-27 cells (**Figures 6A, B**) (**Supplementary Table S1**). Protein–protein interaction (PPI) network analysis was further performed to explore the potential functional relationships among the DEGs. The resulting network revealed extensive interactions among a subset of DEGs, with HSPA5 and HSP90AA1 occupying relatively central positions and showing multiple connections with genes including DNAJC3, DNAJB9, SEC61A1, HERPUD1, SEL1L, ERO1B, and HSPA13 (**Figure 6C**). Notably, many of these highly interconnected genes are involved in endoplasmic reticulum protein processing and cellular stress responses, consistent with the functional enrichment results. These findings suggest that miR-132-3p overexpression may induce coordinated transcriptional alterations involving interconnected protein-processing and stress-response networks in HGC-27 cells.

**Figure 6:**
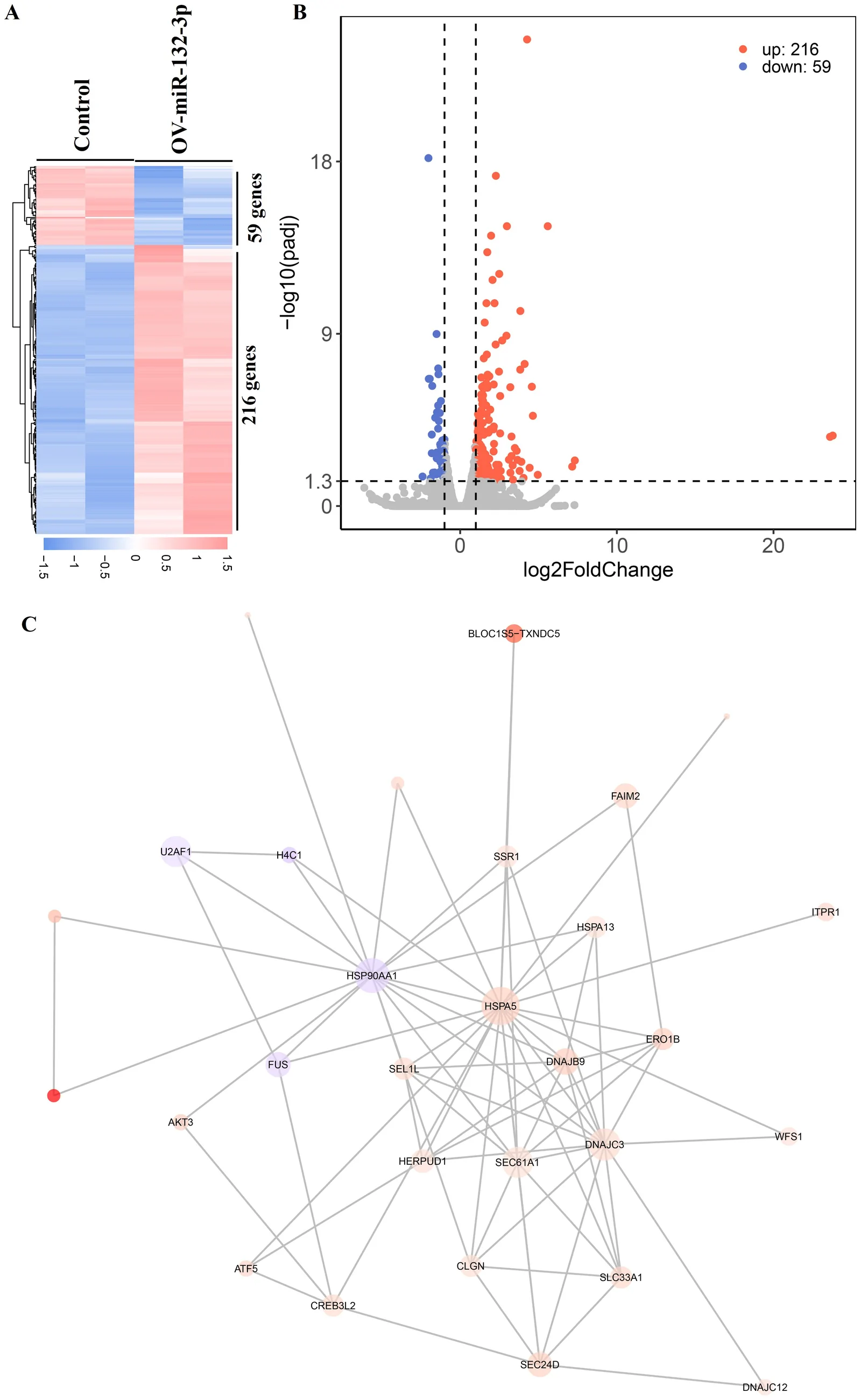
Transcriptional alterations in HGC-27 cells after miR-132-3p overexpression. **(A)** Heatmap of DEGs. **(B)** Volcano plot of DEGs **(C)** PPI network analysis of DEGs.

GO analysis illustrated that these downregulated DEGs were primarily associated with RNA binding, proton transmembrane transporter activity, oxygen transport, oxygen binding, hemoglobin complex, ATP synthesis coupled proton transport (**Figure 7A**). These upregulated DEGs were primarily associated with endoplasmic reticulum, serine-type endopeptidase inhibitor activity, chloride channel activity, calcium ion transport and cell adhesion (**Figure 7B**). KEGG analysis illustrated that these downregulated DEGs were primarily enriched in oxidative phosphorylation, thermogenesis, spliceosome, IL-17 signaling pathway, cAMP signaling pathway and Calcium signaling pathway (**Figure 7C**). These upregulated DEGs were primarily enriched in protein processing in endoplasmic reticulum, protein digestion and absorption, thyroid hormone synthesis, focal adhesion, cAMP signaling pathway, autophagy-animal, apelin signaling pathway and adrenergic signaling in cardiomyocytes (**Figure 7D**).

**Figure 7:**
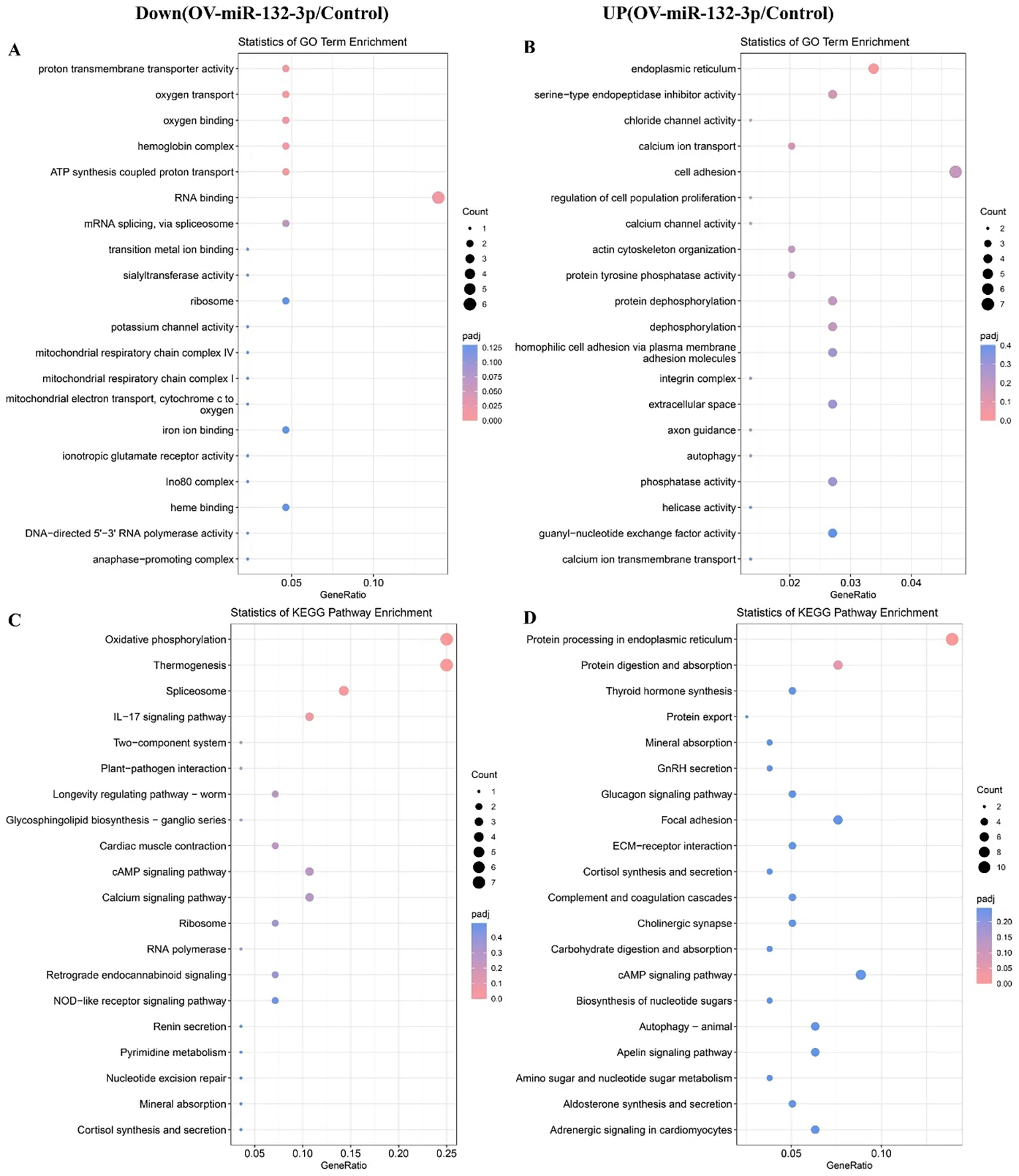
Enrichment analysis of DEGs. **(A)** GO enrichment analysis of downregulated DEGs. **(B)** GO enrichment analysis of upregulated DEGs. **(C)** KEGG enrichment analysis of downregulated DEGs. **(D)** KEGG enrichment analysis of upregulated DEGs.

The GSEA revealed that the main GO functions were structural constituent of ribosome, translation, mRNA splicing via spliceosome, ATP synthesis coupled proton transport, endoplasmic reticulum, endocytosis, potassium ion transport, cellular amino acid metabolic process and calmodulin binding (**Figure 8A**). KEGG analysis illustrated that these DEGs showed enrichment mostly in functions such as the ribosome, oxidative phosphorylation, spliceosome, ATP-dependent chromatin remodeling, salivary secretion, thyroid hormone synthesis, nucleotide excision repair, gastric acid secretion, RNA polymerase and proteasome (**Figure 8B**).

**Figure 8:**
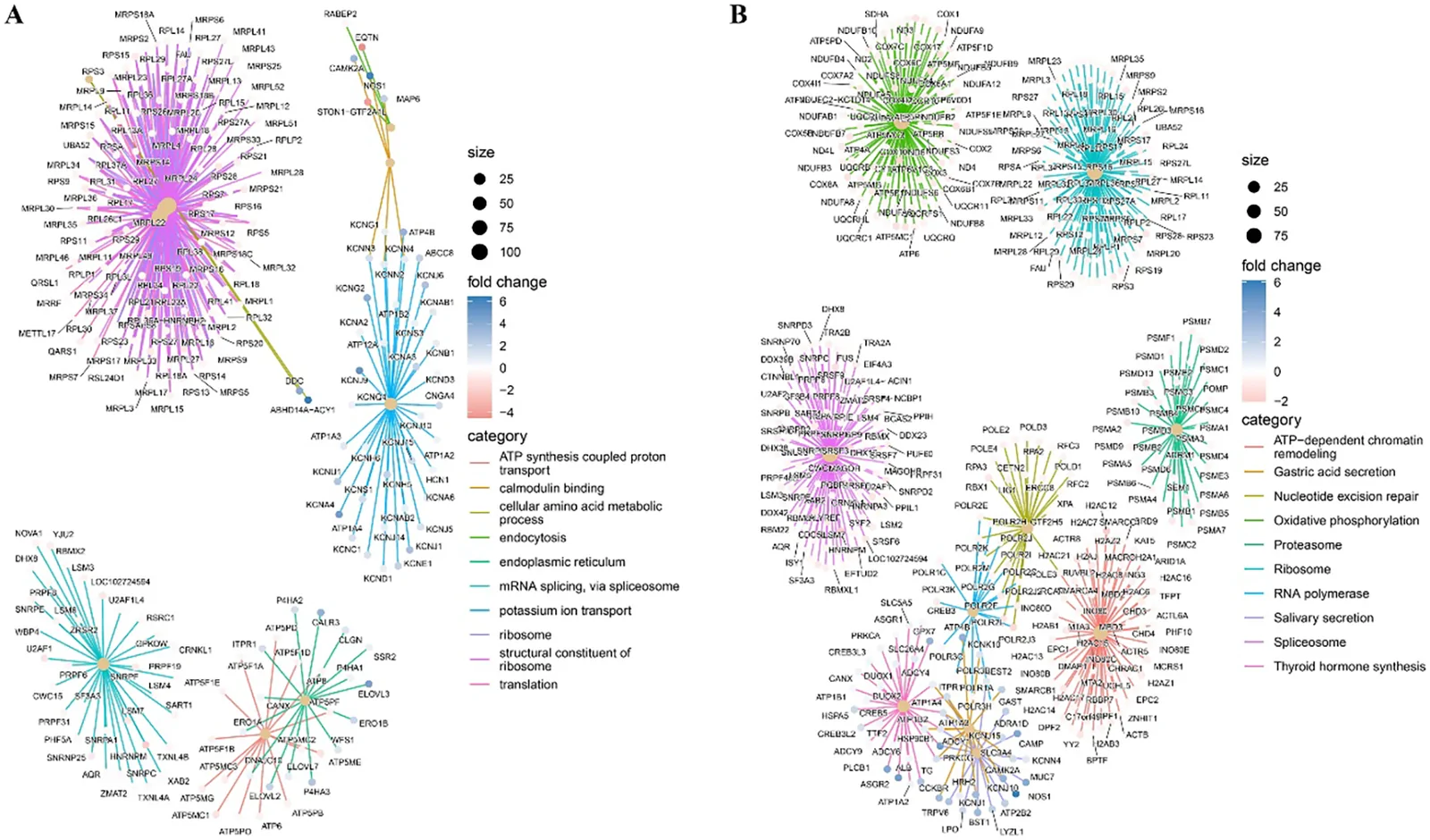
GSEA analysis of DEGs. **(A)** GO/GSEA analysis of DEGs. **(B)** KEGG/GSEA analysis of DEGs.

Collectively, these transcriptomic analyses indicate that miR-132-3p overexpression induces broad transcriptional remodeling in HGC-27 cells, particularly affecting endoplasmic reticulum protein processing and stress responses, mitochondrial energy metabolism, RNA processing, and calcium-related signaling pathways.

### 3.8 miR-132-3p regulates ferroptosis-related genes in HGC-27 cells

To identify ferroptosis-related transcriptional alterations associated with miR-132-3p overexpression, DEGs between the control and miR-132-3p mimic groups were intersected with ferroptosis-related genes obtained from FerrDb. This analysis identified 12 overlapping ferroptosis-related DEGs, including 11 upregulated genes and one downregulated gene (**Figure 9A**). Heatmap and volcano plot analyses further showed that BLOC1S5-TXNDC5, HNF4A, ETV4, WIPI1, HSPA5, NDRG1, IDH1, SAT1, HERPUD1, ENPP2, and HILPDA were upregulated following miR-132-3p overexpression, whereas HBA1 was downregulated (**Figures 9B, C**). To independently validate these transcriptomic findings, the expression of the identified ferroptosis-related DEGs was further examined by qRT-PCR in control, miR-132-3p mimic-, and M2-Exo-treated HGC-27 cells. Consistent with the RNA-seq data, miR-132-3p overexpression significantly increased the expression of most of the identified ferroptosis-related genes and reduced HBA1 expression. Notably, M2-Exo treatment induced a largely similar expression pattern (*P* < 0.01) (**Figure 9D**).

**Figure 9:**
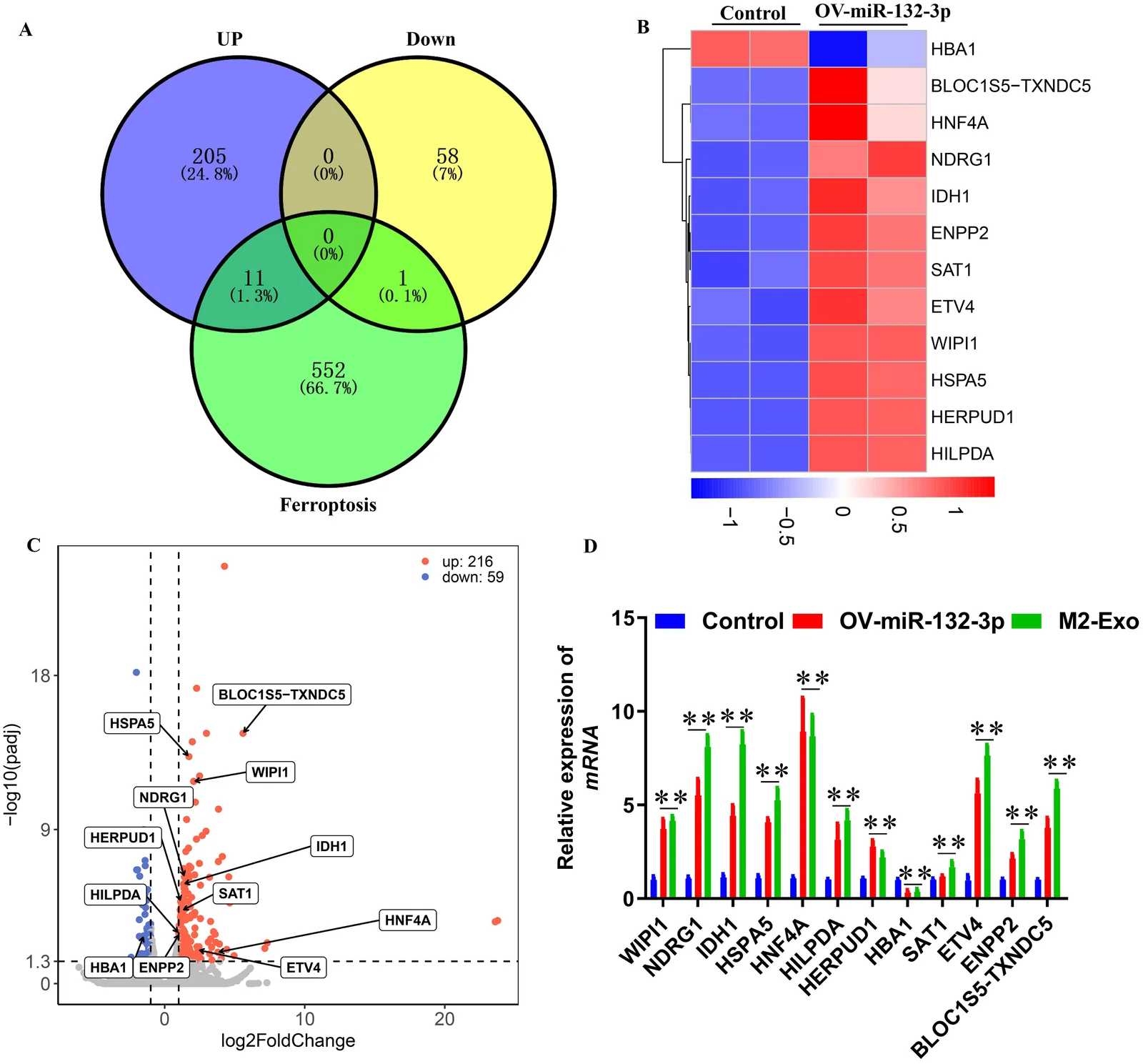
Effects of miR-132-3p on ferroptosis-related gene expression in HGC-27 cells. **(A)** Venn diagram showing the intersection between RNA-seq-derived DEGs and ferroptosis-related genes obtained from FerrDb. **(B)** Heatmap of the overlapping ferroptosis-related DEGs. **(C)** Volcano plot showing the expression changes of ferroptosis-related DEGs. **(D)** qRT-PCR validation of the ferroptosis-related DEGs in HGC-27 cells. ^**^P < 0.01.

Collectively, these results demonstrate that miR-132-3p overexpression is associated with coordinated alterations in ferroptosis-related gene expression and that M2 macrophage-derived Exos produce a broadly similar transcriptional response in HGC-27 cells.

## 4 Discussion

GC diagnosis is often at an advanced stage owing to its silent onset; by this stage, distant metastasis has typically developed, significantly affecting both treatment outcomes and quality of life (1). GC is a prevalent malignancy affecting public health in China (2). The progression of GC is a complicated process affected by several factors (e.g., genetic predisposition, infection, dietary habits, and environmental pollutants). Key contributors include the driving genes of malignant GC, the presence of malignant ascites, and chronic inflammation induced by *Helicobacter pylori* infection (15, 16). Investigations have indicated that dysregulated expression of miRNAs participate in the pathogenesis and progression of GC, with associations observed in tumor growth, metastasis, drug resistance, immune tolerance, and modifications within the GC immune microenvironment. These findings suggested that miRNAs may act as early diagnostic markers and potential therapeutic targets for GC (17). TAMs contribute to GC proliferation and drug resistance by secreting EXOs that carry miRNAs (18). Notably, miR-132-3p is markedly overexpressed in M2 macrophages and is closely related to M2 macrophage polarization (19). Analysis of TCGA data illustrated that miR-132-3p upregulation was detected in GC and exhibits a correlation with tumor stage and grade. Additionally, the miR-132-3p expression was increased significantly in the M2 macrophage-derived EXOs. Consequently, we assessed the miR-132-3p function in promoting GC progression and its association with M2 macrophage-derived EXOs, to clarify the connection between miR-132-3p and the GC progression.

Extracellular vesicles (EVs) are membrane-bound structures that cells release under physiological and pathological conditions, carrying proteins, lipids, nucleic acids, and other bioactive molecules. Based on their mechanisms of secretion, EVs can be categorized into microvesicles, EXOs, apoptotic bodies, and migrating bodies. EXOs secreted by cells are generally 30–150 nm in size and are membrane-bound vesicles developed via the inward budding of the cellular membrane (20). These vesicles contain various essential biomolecules, including four transmembrane protein families (CD9/63/81), heat shock proteins, growth factors, major histocompatibility complex (MHC I/MHC II), adhesion proteins, miRNAs, enzymes, lipids, and genetic material. Among these, CD9, CD63, CD81, HSP70, and Alix are specific markers of EXOs (21). EXO-mediated intercellular communication regulates normal physiological functions and contributes to pathological mechanisms in various diseases, including cancer, making EXOs potential targets for tumor diagnosis and therapy (22, 23).

Tumor microenvironment (TME) comprises heterogeneous immune and stromal cell populations, cytokines, extracellular vesicles, and tumor-derived factors that collectively regulate tumor progression and therapeutic responses (24). Tumor-associated macrophages, particularly M2-like macrophages, can contribute to an immunosuppressive microenvironment through both soluble mediators and exosome-mediated intercellular communication and remodeling an immunosuppressive microenvironment has been shown to affect the efficacy of immune checkpoint blockade in GC (25). Recently studies have shown that TAMs exchange data with tumor cells via EXOs and have a function in multiple tumor-related processes (26). TAM-derived EXOs can carry miR-513b-5p, which triggers the invasion and migration of GC (5). Furthermore, miR-487a expression in M2 macrophage-derived EXOs is upregulated, and upon transfer to GC cells, it targets T-cell intracellular antigen 1 (TIA1), a cytotoxic granule-correlated RNA-binding protein, thus inducing cell growth and promoting the progression of GC (27). Additionally, miR-21 carried by M2 macrophage-derived EXOs suppresses apoptosis by downexpressing PTEN through stimulation of the PI3K/AKT pathway, leading to an increase in cisplatin (DDP) resistance in GC cells (18). In this study, miR-132-3p expression in M2 macrophage-derived EXOs was significantly elevated, and these EXOs considerably enhanced the aggressiveness of HGC-27 cells. Suppression of miR-132-3p expression significantly inhibited these processes, indicating that the M2 macrophage-derived EXOs’ function in triggering GC progression may be linked to miR-132-3p expression. In GC, miR-132-3p influences cell aggressiveness by targeting KIF21B (kinesin family member 21B) expression (28). Therefore, further investigation into the miR-132-3p effect on GC progression and its potential clinical significance is needed.

Programmed cell death (PCD) is tightly controlled by intricate intracellular and extracellular signaling pathways and has a vital function in numerous biological processes (e.g., mammalian development, homeostasis, and disease progression) (29). Ferroptosis, a form of PCD, differs from traditional cell death mechanisms and is distinguished by intracellular iron overload, severe lipid peroxidation, and extensive mitochondrial ultrastructural alterations (10, 30). Induction of ferroptosis can significantly enhance apoptosis in GC cells and inhibit tumor progression (14). Moreover, ferroptosis in GC cells is triggered by miR-375 by targeting SLC7A11 (Solute Carrier Family 7 Member 11 [xCT]), leading to a reduction of proliferation and decreased stemness maintenance of GC cells (31). Additionally, downregulation or deletion of miR-221-3p upregulates the downstream transcription factor ATF3, which triggers ferroptosis in GC cells. Elevated ATF3 expression inhibits the GPX4 and HRD1 transcription, inhibiting the growth of GC cells, while HRD1 counteracts ferroptosis via facilitating the ACSL4 ubiquitination and degradation (13). Moreover, tumor-associated fibroblasts secrete miR-522, which targets ALOX15, preventing lipid ROS overload and thereby suppressing ferroptosis in GC cells, ultimately promoting tumor growth and drug resistance (32). Herein, M2 macrophage-derived EXOs and miR-132-3p significantly reduced intracellular iron ion concentrations in HGC-27 cells, decreased MDA levels, increased GSH content and SOD activity, and upregulated FTH1 and GPX4 expression. These findings suggested that both miR-132-3p and M2 macrophage EXOs can effectively suppress ferroptosis in HGC-27 cells.

The transcriptomic findings further support a potential link between miR-132-3p and ferroptosis-related regulation in GC cells. RNA-seq followed by GO, KEGG, and GSEA analyses revealed that miR-132-3p overexpression induced broad transcriptional remodeling involving mitochondrial energy metabolism, oxidative phosphorylation, endoplasmic reticulum protein processing and stress responses, RNA processing, and calcium-related signaling. These biological processes are closely related to cellular redox homeostasis and metabolic adaptation and may therefore influence the susceptibility of tumor cells to ferroptotic stress (33, 34). Moreover, integration of the RNA-seq-derived DEGs with FerrDb identified 12 ferroptosis-related DEGs. Among these genes, several, including HSPA5 (35), HERPUD1 (36), NDRG1 (37), SAT1 (38) and HILPDA (39), are involved in cellular stress responses, metabolic regulation, or redox homeostasis, further suggesting that miR-132-3p overexpression is accompanied by coordinated remodeling of ferroptosis-associated pathways. Additionally, qRT-PCR confirmed that most of these ferroptosis-related genes exhibited expression changes consistent with the RNA-seq data, and M2-Exo treatment produced a largely similar expression pattern. Together with the observed reductions in intracellular iron and MDA and the increases in GSH, SOD, FTH1 and GPX4, these transcriptomic changes are consistent with a reduced ferroptosis-related phenotype in HGC-27 cells.

Notably, the PPI network analysis identified HSPA5 as a relatively central node among the DEGs. A previous study has shown that HSPA5 can enhance the expression and activity of GPX4, thereby attenuating lipid peroxidation and protecting glioma cells from dihydroartemisinin-induced ferroptosis (40). Another study also demonstrated that HSPA5 alleviated erastin-induced GPX4 depletion, suppressed ferroptosis, and promoted colorectal cancer cell growth both *in vitro* and *in vivo* (41). According to recent studies, LIFR is frequently downregulated in hepatocellular carcinoma tissues. Loss of LIFR could promote liver tumorigenesis and confer resistance to drug-induced ferroptosis in mice (42). In addition, LIFR knockdown has been shown to promote GC cell survival, migration, invasion, and glycolysis while suppressing apoptosis (43, 44). Our transcriptomic analysis showed that miR-132-3p overexpression was associated with reduced LIFR expression in HGC-27 cells. Consistently, Western blot analysis further demonstrated that both miR-132-3p overexpression and treatment with M2 macrophage-derived exosomes decreased LIFR protein expression in HGC-27 cells. Collectively, these findings suggest that HSPA5 and LIFR may represent an important candidate mediator linking miR-132-3p to ferroptosis-related regulation in gastric cancer cells. Nevertheless, these data primarily establish an association between miR-132-3p, M2 macrophage-derived Exos, and ferroptosis-related transcriptional remodeling, and further mechanistic studies are required to determine which of these candidate genes or pathways directly mediate the observed phenotype.

Several limitations of this study should be acknowledged. First, although M2 macrophage-derived Exos and miR-132-3p overexpression produced largely similar effects on the malignant phenotypes and ferroptosis-related alterations of HGC-27 cells, we did not perform rescue experiments involving selective depletion or restoration of miR-132-3p in M2 macrophage-derived Exos. Therefore, the present findings support an association between exosomal miR-132-3p and ferroptosis-related regulation rather than establishing a direct causal relationship. Second, the direct downstream targets of miR-132-3p were not experimentally identified. In particular, although miR-132-3p overexpression was accompanied by decreased LIFR protein expression, direct binding of miR-132-3p to the 3′UTR of LIFR was not validated. Thus, LIFR should currently be considered a candidate downstream molecule rather than a confirmed direct target of miR-132-3p. Third, most functional experiments were performed in a single GC cell line, HGC-27, which may limit the generalizability of the findings. Validation in additional GC cell lines with distinct molecular backgrounds, as well as *in vivo* and clinical samples, will be required. Fourth, although RNA-seq, functional enrichment, PPI network analysis, and integration with FerrDb identified several pathways and ferroptosis-related candidate genes associated with miR-132-3p overexpression, these transcriptomic findings remain largely hypothesis-generating. Further pathway-specific manipulation and functional rescue experiments are needed to determine which candidate genes and signaling networks directly mediate the observed ferroptosis-related and malignant phenotypes. These limitations should be considered when interpreting the mechanistic and potential clinical implications of the present findings.

## 5 Conclusion

In summary, miR-132-3p was markedly enriched in M2 macrophage-derived exosomes. Both M2-Exo treatment and miR-132-3p overexpression promoted malignant phenotypes in gastric cancer cells and were accompanied by reduced ferroptosis-related changes. These findings suggest that exosomal miR-132-3p may contribute to the regulation of ferroptosis and aggressive behavior in gastric cancer cells. However, the present data do not establish a direct causal mechanism, and further studies are required. Therefore, M2 macrophage-derived exosomal miR-132-3p may represent a potential biomarker reflecting tumor–microenvironment interactions and may also provide a candidate target for therapeutic strategies aimed at restoring ferroptosis susceptibility in gastric cancer.

## Data Availability

The RNA-seq data used in this study was deposited in the Science Data Bank (DOI: https://doi.org/10.57760/sciencedb.012v4). The online version contains the RNA-seq data, available at: https://www.scidb.cn/en/s/aeqYRj. Other data supporting the findings of this study are available from the corresponding author upon reasonable request.
